# Adenosine A1 Receptor Agonist 2-chloro-N6-cyclopentyladenosine and Hippocampal Excitability During Brain Development in Rats

**DOI:** 10.3389/fphar.2019.00656

**Published:** 2019-06-14

**Authors:** Petr Fabera, Martina Parizkova, Libor Uttl, Katerina Vondrakova, Hana Kubova, Grygoriy Tsenov, Pavel Mares

**Affiliations:** ^1^Department of Developmental Epileptology, Institute of Physiology, Czech Academy of Sciences, Prague, Czechia; ^2^Department of Neurology, Second Faculty of Medicine, Motol University Hospital, Charles University, Prague, Czechia; ^3^National Institute of Mental Health, Klecany, Czechia; ^4^Department of Neurosciences, Biomedicine and Movement Sciences, University of Verona, Verona, Italy

**Keywords:** adenosine receptor, agonist, development, hippocampus, epileptic afterdischarges, rat

## Abstract

**Objective:** The adenosinergic system may influence excitability in the brain. Endogenous and exogenous adenosine has anticonvulsant activity presumably by activating A1 receptors. Adenosine A1 receptor agonist 2-chloro-N6-cyclopentyladenosine (CCPA) may thus bolster anticonvulsant effects, but its action and the number of A1 receptors at different developmental stages are not known.

**Methods:** Hippocampal epileptic afterdischarges (ADs) were elicited in 12-, 15-, 18-, 25-, 45-, and 60-day-old rats. Stimulation and recording electrodes were implanted into the dorsal hippocampus. The A1 receptor agonist 2-chloro-N6-cyclopentyladenosine (CCPA, 0.5 or 1 mg/kg) was administered intraperitoneally 10 min before the first stimulation. Control animals were injected with saline. All rats were stimulated with a 2-s series of 1-ms biphasic pulses delivered at 60 Hz with increasing stepwise intensity (0.05–0.6 mA). Each age and dose group contained 9–14 animals. The AD thresholds and durations were evaluated, and the A1 receptors were detected in the hippocampus in 7-, 10-, 12-, 15-, 18-, 21-, 25-, 32-, and 52-day-old rats.

**Results:** Both CCPA doses significantly increased hippocampal AD thresholds in 12-, 15-, 18-, and 60-day-old rats compared to controls. In contrast, the higher dose significantly decreased AD threshold in the 25-day-old rats. The AD durations were significantly shortened in all age groups except for 25-day-old rats where they were significantly prolonged. A1 receptor expression in the hippocampus was highest in 10-day-old rats and subsequently decreased.

**Significance:** The adenosine A1 receptor agonist CCPA exhibited anticonvulsant activity at all developmental stages studied here except for 25-day-old rats. Age-related differences might be due to the development of presynaptic A1 receptors in the hippocampus.

## Introduction

The roles of exogenous and endogenous purine nucleosides in the central nervous system are currently under intense investigation (Ipata, 2011; Ribeiro et al., 2016; Fumagalli et al., 2017). Adenosine is an endogenous neuromodulator implicated in the pathophysiology of various neurological diseases including epilepsy, stroke, chronic pain, and dementia (Sollevi, 1997; Boison, 2005; Arosio et al., 2016). The physiological functions of this nucleoside in the nervous system are exerted by binding to G-protein coupled receptors, which are classified into four categories: A1, A2A, A2B, and A3 (Zhou et al., 1992). The A1 and A2A receptors are both present in the brain; inhibitory A1 receptors are ubiquitous with the highest concentrations in the hippocampus and neocortex (Rivkees et al., 1995). The excitatory A2A receptors are mainly, but not exclusively, in the striatum (Wojcik and Neff, 1983). A1 and A2A receptors are mostly located in the excitatory glutamatergic synapses (Rebola et al., 2005), but both are also found in inhibitory GABAergic synapses (Cunha and Ribeiro, 2000).

Adenosine exerts its modulatory role through activation of A1 receptors by suppressing synaptic activity (Janusz and Berman, 1993) and inhibiting neurotransmitter release (Cunha et al., 1994). These effects are mainly due to activation of presynaptic A1 receptors, leading to the inhibition of glutamate release (Fastbom and Fredholm, 1985). However, adenosine also constitutively activates A2A receptors, which allow in *in vivo* experiments plasticity (Rebola et al., 2003a; Costenla et al., 2011).

The anticonvulsant activities of adenosine A1 analogues in the hippocampus have been demonstrated in adult rats (Ault and Wang, 1986). Based on these findings, adenosine neuromodulation in the immature brain should also be considered. Previous experiments with drugs affecting adenosine receptors demonstrated that the role of adenosine differs according to the level of maturation (Mares, 2014). The immature brain is more prone to seizure activity than the adult brain (Moshe, 2010), and excitability of the hippocampal structure is higher than that in the developing neocortex (Abdelmalik et al., 2005).

Our previous experiments revealed anticonvulsant activities of adenosine analogues in two seizure models: pentetrazol-induced convulsions and cortical epileptic afterdischarges (ADs). Epileptic afterdischarges (ADs) elicited by hippocampal stimulation is a model that is routinely used in our laboratory (Zavala-Tecuapetla et al., 2014). The hippocampus is the most frequently stimulated brain area (Gorter et al., 2016), and hippocampal ADs are a model of complex partial seizures in temporal lobe epilepsy (Kandratavicius et al., 2014). Temporal lobe epilepsy is characterized by spontaneous seizures originating from a spatially restricted region of neuronal hyperexcitability including the hippocampus. Temporal lobe epilepsy represents most drug-resistant cases of human epilepsies (Aicardi and Shorvon, 1997). In addition, more than half of human epilepsies begin in infancy and early childhood (Johnston, 2004), and therefore, developmental data on hippocampal seizures and potential anticonvulsant drugs are of major interest.

The development of A1 receptors might be related to the age dependency of epilepsy. Nevertheless, developmental changes in the expression of A1 receptors and hippocampal excitability in rats have not yet been directly correlated. We studied the activity of a specific A1 receptor agonist, 2-chloro-N6-cyclopentyladenosine (CCPA), on hippocampal excitability and possible changes in this activity with age. CCPA is the most potent and selective A1 receptor ligand characterized in rat brain (Klotz et al., 1989). Rats in developmental stages corresponding to the human perinatal period, preschool and school-age children and young adults, were selected for CCPA administration (six age groups: 12, 15, 18, 25, 45, and 60 days old). These data were correlated to biochemical analysis (nine age groups: 7, 10, 12, 15, 18, 21, 25, 32, and 52 days old).

## Material and Methods

The experimental protocol was approved by the Animal Care and Use Committee of the Institute of Physiology, Czech Academy of Sciences and is consistent with the Animal Protection Law of the Czech Republic and European Community Council directives 86/609/EEC. The Institute of Physiology possessed an National Institutes of Health (NIH) Statement of Compliance with Standards for Humane Care and Use of Laboratory Animals (# A5820-01 valid until 1/31/2019).

### Animals

Experiments were performed using 252 male albino Wistar rats (bred at the Institute of Physiology, Czech Academy of Sciences, Prague) at postnatal (P) days P7, P10, P12, P15, P18, P25, P32, P45, P52, and P60. The day of birth was counted as P0, and weaning took place at P21. Animals were housed in a controlled environment (12:12 h light:dark cycle, temperature 22 ± 1°C, humidity 50–60%) with *ad libitum* access to food and water.

### Surgery

**Figure 1:**
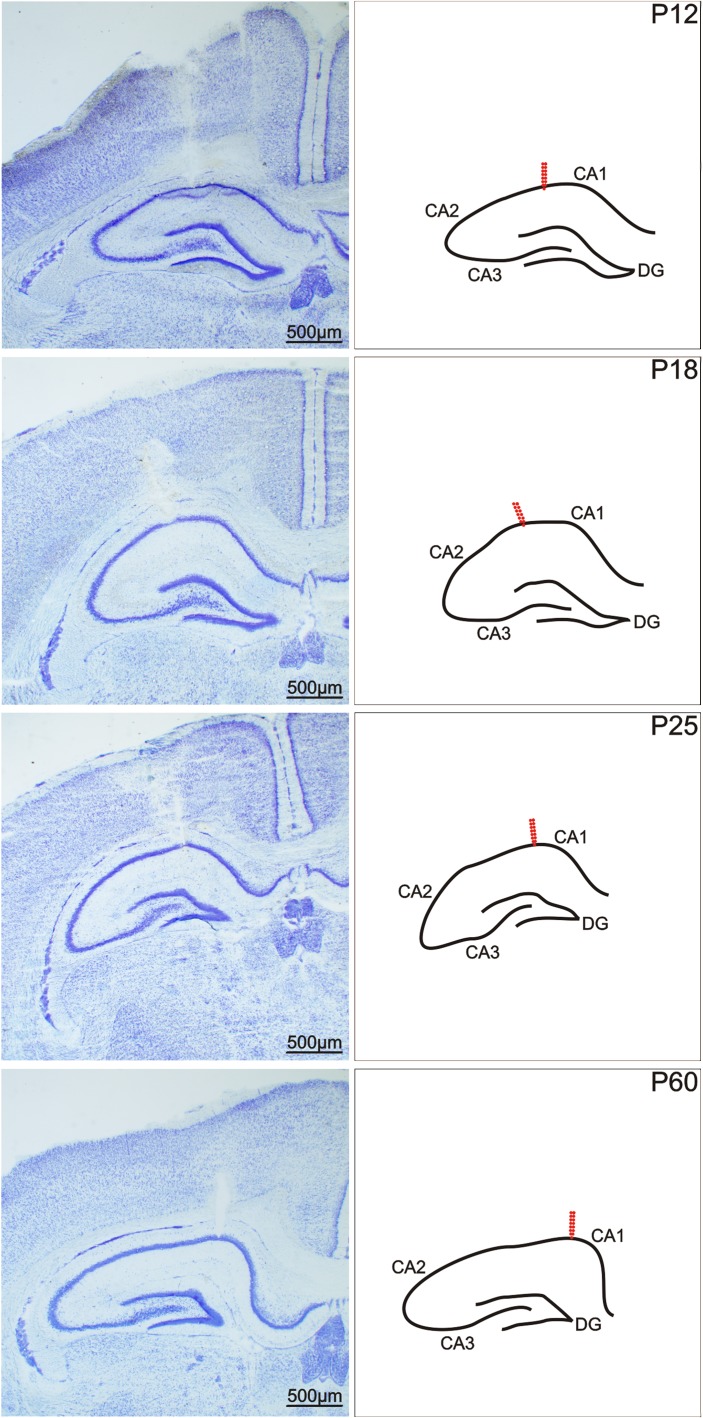
Coronal sections of a rat hippocampus after electrical stimulation of the CA1 regions. From left to right: In Nissl-stained sections, in a schematic imagery. From top to bottom: 12-, 18-, 25-, and 60-day old rats. Arrows show the location of the registration electrodes. Scale bar, see inset.

Surgery was performed under isoflurane anaesthesia. A deep hippocampal stimulation electrode (Plastics One, Roanoke, VA, USA) was implanted stereotaxically into the right dorsal hippocampus, and a recording electrode was implanted into the left dorsal hippocampus at coordinates AP (anteroposterior) -3.0 mm, L (lateral) +2.8 mm, D (dorsal) +3.0 mm, D +3.0 mm for young adult rats; the coordinates were recalculated for immature animals based on the bregma–lambda distance. After the stimulation procedures, the animals were sacrificed and the location of the electrodes was histologically verified in Nissl-stained sections of hippocampus (**Figure 1**). Two flat silver recording electrodes were placed epidurally over the sensorimotor cortex of the left and right hemispheres at coordinates AP +1 mm, L +2 mm. The reference and ground electrodes were placed over the cerebellum. Electrodes were connected to a six-pin connector and fixed to the skull with fast-curing dental acrylic (Duracrol^©^, Dental, Prague). Implantation of electrodes took less than 30 min, at which time the isoflurane anaesthesia was discontinued. The animals were allowed to recover for at least 1 h before the experimental procedures began. Rat pups with immature thermoregulation (up to the end of the third postnatal week) were placed on a pad heated electrically at 34°C during the recovery period as well as during the experiment.

### Stimulation Procedure

Animals were placed individually into plastic boxes and connected to the amplifier and stimulator. An isolated pulse stimulator (model 2100, A-M Systems, Sequim, WA, USA) with constant current output was used. Hippocampal ADs were elicited with a series of biphasic 1-ms pulses applied for 2 s at 60-Hz frequency with stepwise increasing current intensity from 0.05 mA to 0.6 mA. Seven series of 2-s stimulations were applied over 20-min intervals.

### Recording

A TDT Open Project Program (Tucker-Davis Technologies, Alachua, FL, USA) was used to record the electrophysiologic signals. All signals were amplified (Pentusa Base Station, Tucker-Davis Technologies) and digitized at 1 kHz.

### Drugs

The A1 adenosine receptor agonist CCPA was administered intraperitoneally at doses of 0.5 mg/kg or 1 mg/kg. The drug was purchased from Tocris Biosciences (Bristol, UK), and the doses were selected based on prior work (Mares, 2014). All age and dose groups comprised 9–14 animals. CCPA was placed in suspension with a drop of Tween 80 and diluted with saline immediately before the experiment to obtain a final concentration of 1 mg/ml. The control group comprised rats injected with a similar volume of saline. Animals were injected with CCPA or saline 10 min prior to the start of the stimulation procedure.

### Bioanalytical Analysis

Western blot analysis was performed to detect changes in A1 receptors at the nine ages (7, 10, 12, 15, 18, 21, 25, 32, and 52 days). Hippocampal tissue from 54 rats (6 animals/group) was collected. The tissue was frozen and stored at −80°C before analysis. All mixed samples were prepared from six animals with a glass homogenizer with a power-driven Teflon pestle (Helidolph—RZR2021) with 10 mM PBS (pH 7.4) at a 1:4 ratio and protease inhibitor cocktail (# P8340, Sigma-Aldrich, St. Louis, MO, USA). The homogenates were centrifuged (#120951, Sigma-Aldrich 2-16 PK) at 1,000*g* for 10 min at 4°C, and the supernatant was collected.

A small volume of the hippocampus lysate was used for quantification of the protein concentration by Lowry’s method (Lowry et al., 1951) with Peterson’s modification (Peterson, 1997). Before electrophoresis, the samples were mixed at a 1:2 ratio with Laemmli loading buffer (#161-0737, Bio-Rad, Hercules, CA, USA) and heated for 20 min at 70°C. Stain-free gradient gels (# 567-8104, Bio-Rad) were used for protein separation and protein labeling by binding of the trihalo compound to tryptophan residues. After electrophoresis (300 V, 270 mA, 23 min), all gels were activated and visualized with UV light for 5 min by ChemiDoc^™^ Touch Imaging System (Bio-Rad). The samples were subsequently transferred to nitrocellulose membranes (#170-4271, Bio-Rad) using a Trans-blot Turbo apparatus (Bio-Rad). The quality of transfer and volume of protein on the membrane was determined by a ChemiDoc^™^ Touch Imaging System (Bio-Rad). Membranes were blocked in 5% non-fat milk in Tris-buffered saline (TBS) for 1 h at room temperature and were then incubated overnight at 6°C with primary antibodies anti-A1R 1:3,000 (PA1-041A, Thermo Fisher Scientific, Waltham, MA, USA).

The following day, the membranes were washed three times for 10 min in TBS and then incubated for 1 h at room temperature with secondary antibody 1:30,000 (#211-032-171, Jackson ImmunoResearch Laboratories, West Grove, PA, USA) for 1 h at room temperature and washed in TBS as described above. The chemiluminescent substrate (Supersignal WestFemto, #34096, Thermo Scientific) was used to visualize the protein with the ChemiDoc^™^. The bands were detected and analyzed with ImageLab software (Bio-Rad). Stain-free images of total protein were used to normalize the target protein as a loading control (Colella et al., 2012). Western blots were normalized with stain-free technology that is comparable with other total protein staining methods (PonceauS, Coomassie Blues, etc) (Colella et al., 2012).

### Statistics

Statistical evaluation of the electrophysiologic data was performed with SigmaStat^©^ software (SYSTAT Inc., San Jose, CA, USA). This program started with a test of the distribution of the data and recommended a parametric or nonparametric test based on the results. The duration of ADs in individual age and dose groups was evaluated with a one-way, repeated-measures analysis of variance (RM ANOVA). Threshold intensities for hippocampal epileptic ADs in the three treatment groups were compared with a one-way ANOVA. Subsequent pairwise comparisons were performed using the Holm–Sidak test. A *p* < 0.05 was considered statistically significant.

The chemiluminescence of the target proteins and stain-free images of total protein was analyzed in ImageLab (Bio-Rad). Statistical analysis was performed in Statistica 9 (Statsoft, 2009), and the graphs were created in Prism (version 5; GraphPad, La Jolla, CA, USA). Differences between the nine age groups were evaluated with one-way ANOVA and subsequently with a *post hoc* Tukey test to determine the statistical significance (*p* < 0.05). Data are presented as mean gray value ± SEM.

## Results

### Threshold Intensity

**Figure 2:**
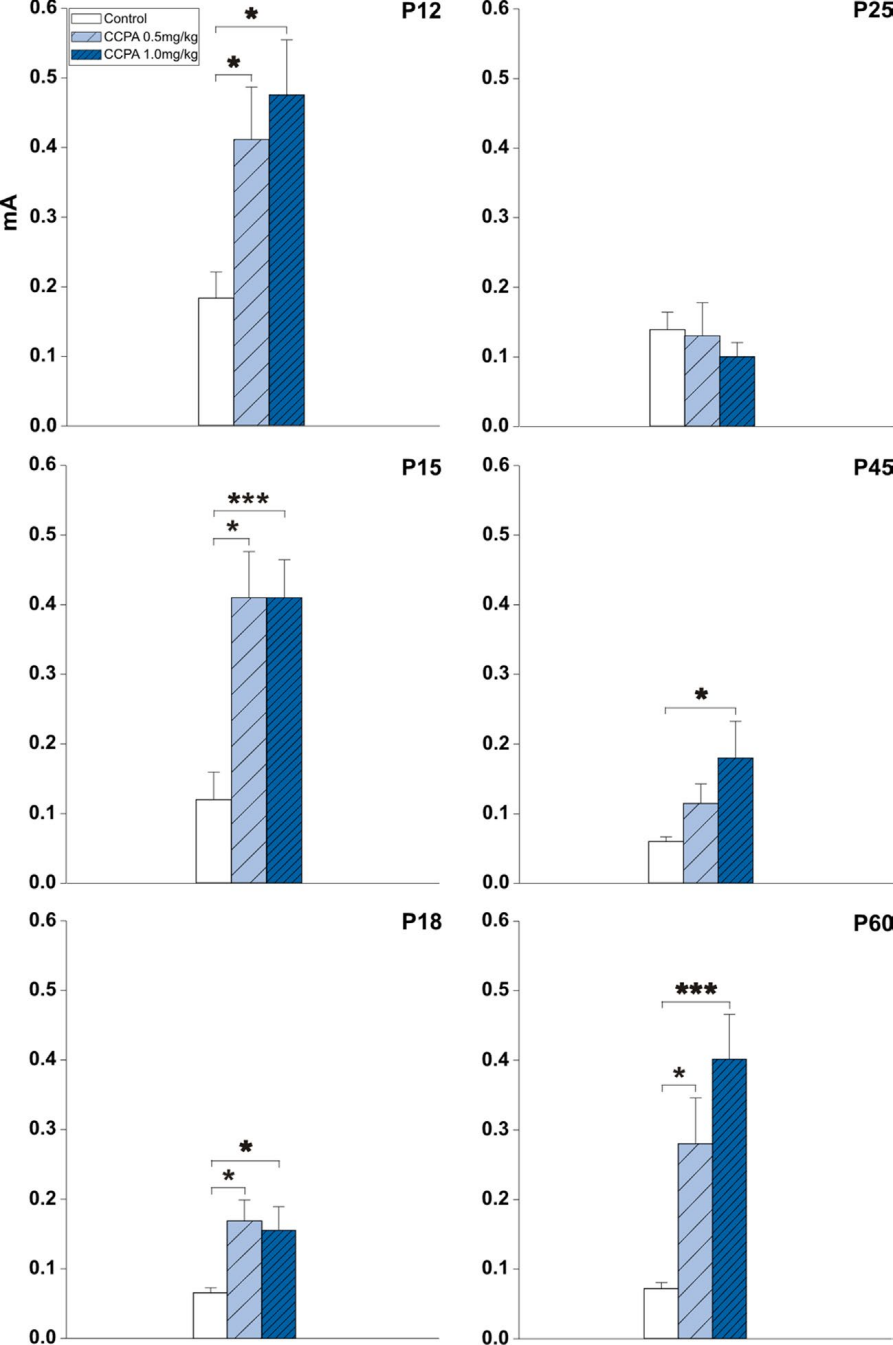
Effects of adenosine A1 receptor agonist 2-chloro-N6-cyclopentyladenosine (CCPA) on threshold intensity for elicitation of hippocampal afterdischarge (mean + SEM) in five age groups. From top to bottom: 12-, 15-, 18-, 25-, 45-, and 60-day old rats. Ordinates: Intensity of stimulation in mA. White columns—control rats injected after the first AD with saline, obliquely stripped columns—animals given 0.5 or 1.0 mg/kg of CCPA (see inset). One or three asterisks denote significant difference from corresponding control afterdischarges (ADs) with *p*-values <0.05 or <0.001, respectively.

A threshold is the lowest current intensity necessary for a hippocampal AD to occur. Age-dependent changes in the threshold intensity of hippocampal ADs were detected in both control and drug pre-treated rats. One-way ANOVA revealed changes in treatment interaction as a function of age for the hippocampal AD threshold in animals administered CCPA. Elicitation of hippocampal ADs in 12-, 15-, and 18-day-old CCPA pre-treated rats required significantly higher current intensities in both dose groups versus age-matched controls. A similar effect was observed in P45 rats only after the higher (1 mg/kg) dose of CCPA. The oldest group of rats (60 days old) exhibited a significantly higher threshold intensity after either dose of CCPA. In contrast, the higher dose of CCPA in 25-day-old rats did not result in any significant changes in the AD threshold intensity (**Figure 2**).

### Duration of Hippocampal Afterdischarges

**Figure 3:**
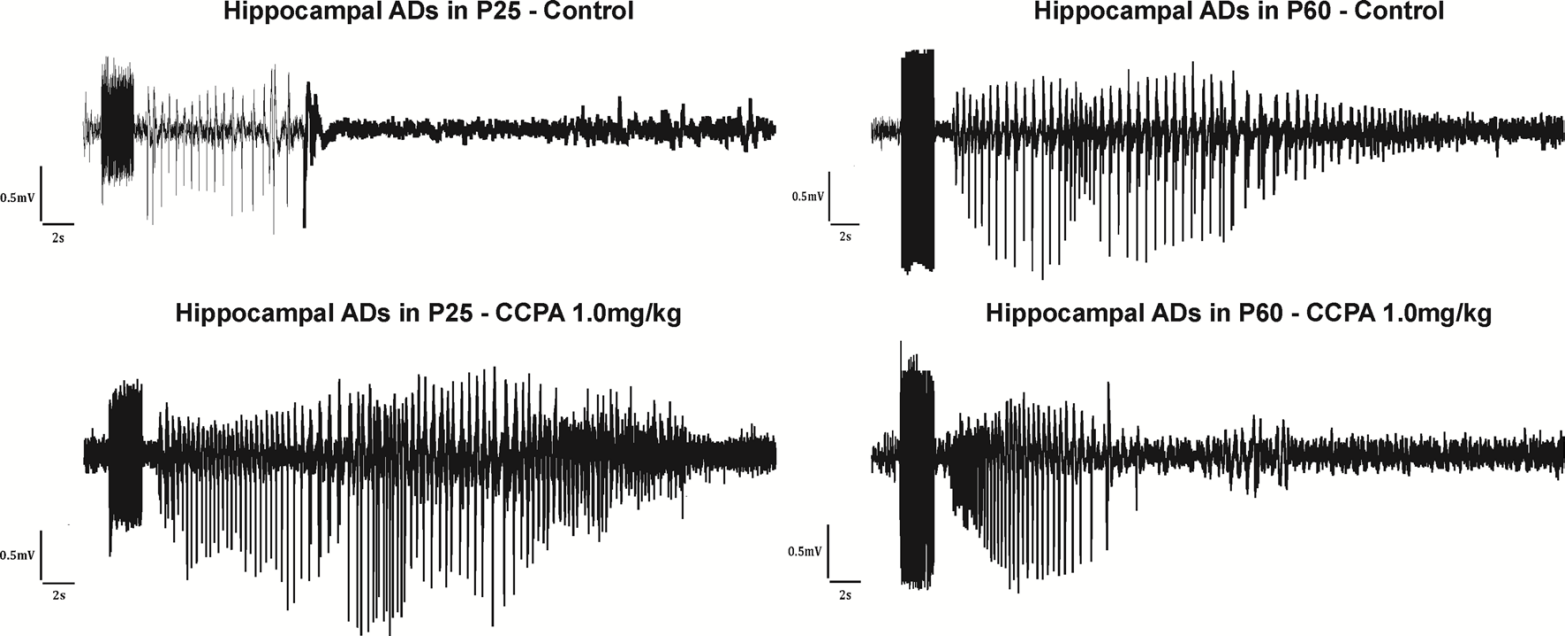
Hippocampal ADs elicited by electrical stimulation in 25- and 60-day-old rats accompanied by epileptic automatisms (wet dog shakes). From top to bottom: control animals, animals after the administration of CCPA at 1 mg/kg. From left to right: 25- and 60-day-old rats.

The total duration of the hippocampal ADs was measured. With slightly suprathreshold intensities of stimulation current, ADs were formed by a fast polyspike (PS), large delta, and/or sharp theta waves (Kotloski et al., 2002) usually followed by a pattern of low-amplitude fast oscillation (Bragin et al., 1997) (**Figure 3**). Stimulation of the hippocampus was associated with stereotypical behavior (automatisms) characteristic for seizures originating in the limbic structures. Wet dog shakes (WDS) were observed at the end of an electroclinical seizure or at the moment when the ADs stopped. WDS could be taken as a sign of hippocampal involvement (Frush and McNamara, 1986). In immature rats, WDS were not regularly present during the first two postnatal weeks but did occur with maturation (Veliskova et al., 1988).

**Figure 4:**
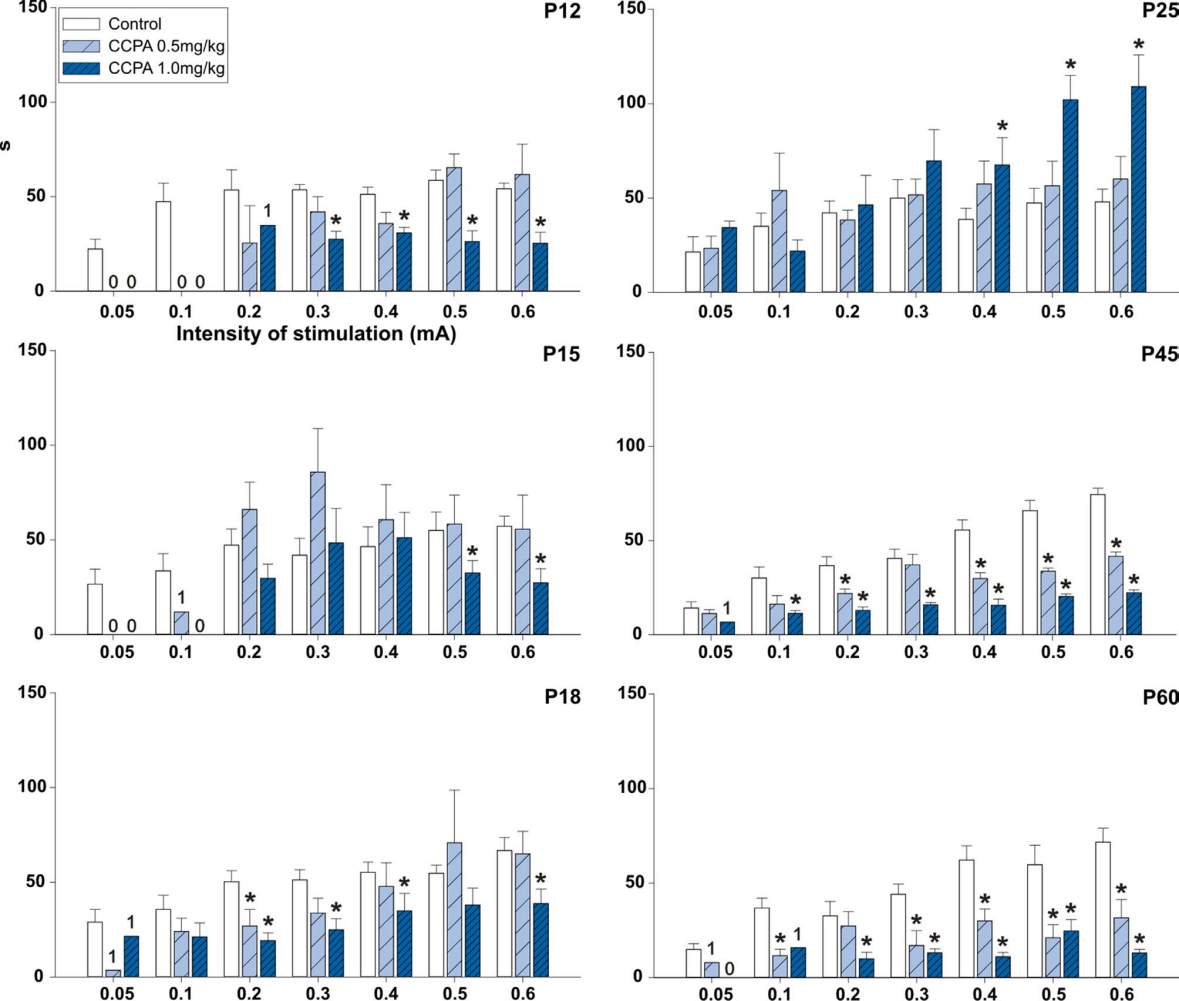
Effects of adenosine A1 receptor agonist CCPA on absolute duration of hippocampal ADs (mean + SEM) in six age groups. From top to bottom: 12-, 15-, 18-, 25-, 45-, and 60-day-old rats. Abscissae: stepwise intensity from 0.05 mA up to 0.6mA; ordinates: absolute duration of ADs in seconds. White columns—control rats injected after the first AD with saline, obliquely stripped columns—animals given 0.5 or 1.0 mg/kg of CCPA (see inset). Asterisks denote significant differences versus the control ADs with *p*-values of <0.05 or <0.001, respectively. Numbers denote one, two, or no animal with elicited AD in each group.

In the 12-day-old (*p* = 0.054–0.279; *F* = 0.555–4.967) and 15-day-old groups (*p* = 0.074–0.287; *F* = 1.304–3.842), 0.5 mg/kg CCPA did not change the AD duration at any stimulation intensity. In 18-day-old rats, 0.5 mg/kg CCPA significantly decreased the AD duration only at the 0.2-mA stimulation intensity (*p* = 0.002; *F* = 15.413). In contrast, the 1-mg/kg dose significantly shortened the AD duration in the 12-day-old (*p* = 0.014–0.459; *F* = 0.653–20.626), 15-day-old (*p* = 0.017–0.203; *F* = 1.883–20.508), and 18-day-old rats (*p* = 0.023–0.232; *F* = 1.553–6.368). This effect was more pronounced in the 12-day-old rats—it appeared after stimulation at 0.3- to 0.6-mA intensity (*p* = 0.014–0.034; *F* = 7.662–20.626), whereas a significant decrease in the AD duration was present only after stimulation at the 0.5- and 0.6-mA intensity in the 15-day-old animals (*p* = 0.017–0.022; *F* = 18.552–20.508). The 18-day-old rats demonstrated a significant decrease in AD duration after stimulation intensities of 0.2–0.6 mA (*p* = 0.023–0.048; *F* = 5.087–6.368) except for the 0.5-mA intensity (*p* = 0.101; *F* = 2.950). In 25-day-old rats, 1.0 mg/kg CCPA significantly prolonged the AD duration versus controls. The prolonged ADs occurred at stimulation intensities of 0.4–0.6 mA (*p* = 0.009–0.047; *F* = 4.474–8.284). In the oldest groups of rats, both CCPA (0.5 mg/kg and 1.0 mg/kg) doses led to a significant decrease in the hippocampal AD duration at stimulation intensities of 0.1–0.6 mA (*p* = 0.005–0.119; *F* = 2.224–13.236 and *p* = 0.007–0.023; *F* = 7.877–16.431). The significant decrease at 0.1-mA and 0.3-mA intensity in P45 (*p* = 0.075; *F* = 4.374 and *p* = 0.242; *F* = 1.633) and 0.2-mA intensity (*p* = 0.119; *F* = 2.224) in P60 group were seen only at animals treated with 0.5 mg/kg CCPA. Changes between both doses of CCPA were exceptional (**Figure 4**).

### Analysis of A1 Receptor

**Figure 5:**
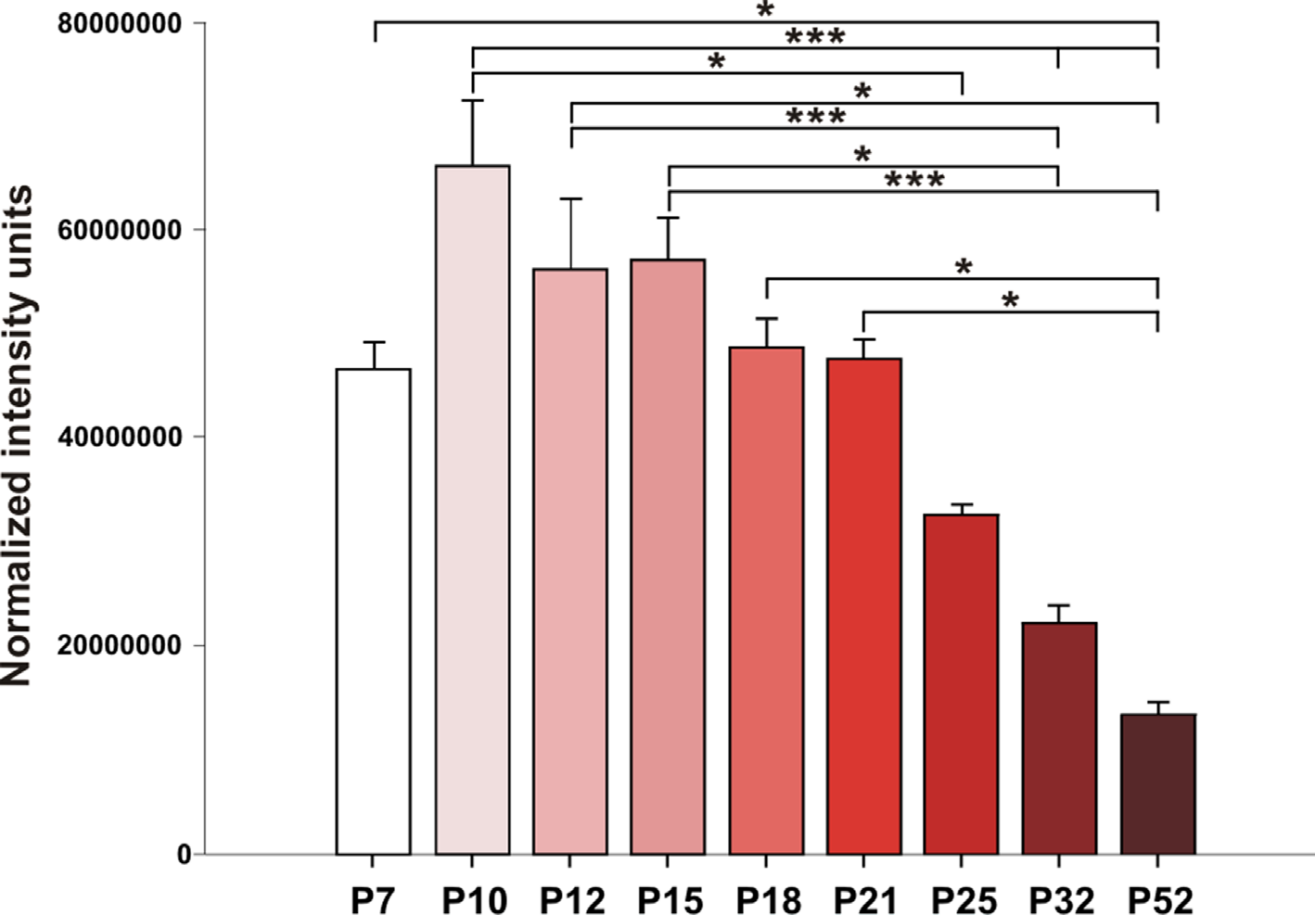
Analysis of A1 receptors (mean gray value + SEM) for nine age groups. Abscissae from left to right: 7-, 10-, 12-, 15-, 18-, 21-, 25-, 32-, and 52-day-old rats; ordinates: normalized intensity units. Asterisks denote significant differences versus the control ADs with *p*-values of <0.05 or <0.001, respectively.

**Figure 6:**
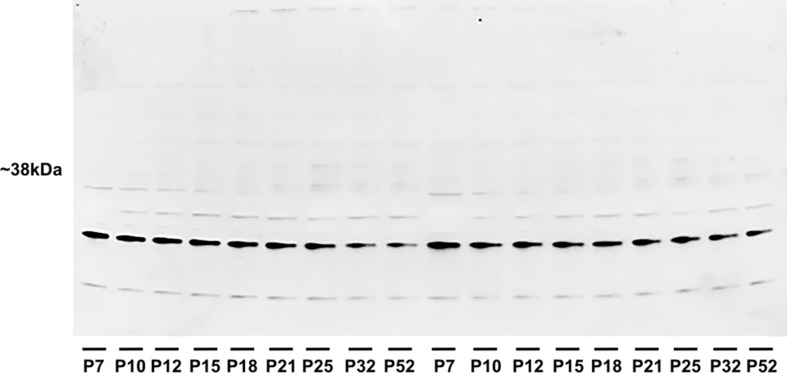
Western blotting of hippocampal homogenates showing the density of adenosine A1 receptor during brain development in rats. Abscissae from left to right: 7-, 10-, 12-, 15-, 18-, 21-, 25-, 32-, and 52-day-old rats in identical replicates.

Hippocampal expression of A1 receptors was highest in 10-day-old rats, and levels in 12-, 15-, and 18-day-old rats tended to be lower. However, statistical significance was reached in P25 animals [*F*(8, 27) = 8.9424, *p* < 0.01] and older animals. Tukey *post hoc* analyses showed a significantly lower expression of A1 receptors in the P32 rats compared to the P12 and P15 groups (*p* < 0.01) as well as in the P52 animals than in the 21-day-old group and younger (*p* < 0.01) (**Figures 5** and **6**).

## Discussion

Adenosine functions as an endogenous brain anticonvulsant *via* the activation of A1 adenosine receptors (Dunwiddie and Masino, 2001). Previously, A1 receptors were shown to control epileptiform discharges in the hippocampal slices of juvenile rats (Tancredi et al., 1998). In this study, we demonstrated for the first time the *in vivo* anticonvulsant activity of the A1 receptor agonist CCPA in the hippocampus of immature rats as well as opposite effect in 25-day-old animals. Adenosine is a homeostatic network regulator that affects different signaling pathways and neurochemical substrates underlying the pathogenesis of temporal lobe epilepsy (Boison and Aronica, 2015). Temporal lobe epilepsy is the most common form of intractable epilepsy and is preferentially associated with functional and morphological alterations of the hippocampus. Hippocampal epileptic ADs represent a model of focal seizures seen in temporal lobe epilepsy (Gorter et al., 2016).

The anticonvulsant effects of CCPA in these experiments were similar to those seen after 2-chloroadenosine and CCPA administration in cortical epileptic ADs (Pometlova et al., 2010; Mares, 2014). In agreement with the loss of effect of CCPA on cortical epileptic afterdischarges in P25 rats (Mares, 2014), the proconvulsant action of an A1 receptor agonist was revealed in 25-day-old rats.

Postnatal changes in expression of adenosine receptors have been described in the rodent brain (Geiger et al., 1984; Johansson et al., 1997). A1 receptor expression increases markedly with progressive maturation (Rivkees, 1995). Moreover, their density (Daval et al., 1991) and function (Descombes et al., 1998) mature from postnatal days 10–15 up to days 25–40. In our experiments, we demonstrated the stepwise decrease of A1 receptor during brain development. Levels in P25 and older rats were significantly lower than the levels in P10–P12 brains. Comparison of *in situ* hybridization studies and receptor labeling revealed notable differences in the patterns of A1 receptor mRNA and A1 receptor expression during development. At birth, A1 receptors are represented mainly in the cortex and subcortical areas (Marangos et al., 1982; Geiger et al., 1984). Age-dependent changes in presynaptic modulation *via* A1 receptors have been described in the rat hippocampus (Jin et al., 1993). A1 receptors are widely distributed at birth (∼10% of adult levels) increasing gradually until adulthood and peaking during the second week of postnatal life (Marangos et al., 1982; Rivkees, 1995; Descombes et al., 1998). Moreover, the affinity and binding capacity of A1 receptor decreased in hippocampus upon aging (Cunha et al., 1995). The differences between age groups with respect to the relationship between the CCPA dose and hippocampal AD duration may indicate a developmental shift in the number and sensitivity of adenosine receptors. Our data support this possibility.

A1 receptors are widely present in different tissues including brain endothelial cells. The expression of A1 and A2 receptors increases cell growth (Clark et al., 2007; Mills et al., 2011). We emphasize that the angiogenesis process is ongoing during the first 4 weeks of postnatal development (Ogunshola et al., 2000). We assume that the decrease in the A1 receptor proteins in P25, P32, and P52 groups is due to the sample preparation procedure. This can explain the differences between our observations and autoradiography studies published previously (Marangos et al., 1982; Rivkees, 1995; Descombes et al., 1998).

A1 receptor agonists act not only postsynaptically but also presynaptically (Cechova et al., 2010). Presynaptic activation of A1 receptors inhibits excitatory amino acid transmission by blocking glutamate release; postsynaptically, adenosine decreases the activation of ionotropic glutamate receptors and voltage-sensitive calcium channels (Klishin et al., 1995) while increasing potassium conductance, resulting in pyramidal cell hyperpolarization (Greene and Haas, 1991).

Nonsynaptic mechanisms should also be considered. Membrane potential measurements indicate that nonsynaptic transmission contributes to the observed synchrony in epileptic ADs (Taylor and Dudek, 1982). A1 receptor agonists may inhibit processes that lead to an increase in nonsynaptic connections. Administration of A1 receptor agonists may increase endogenous extracellular adenosine augmenting the role of nonsynaptic events (Boison, 2005). In addition, a rapid increase in adenosine release was observed during seizure activity induced by electrical stimulation (Schultz and Lowenstein, 1978; Lewin and Bleck, 1981). The increasing levels of endogenous extracellular adenosine after repeated stimulation may increase the threshold for seizure induction (Huber et al., 2001). Moreover, variations in the A1 receptor density after seizures have been demonstrated. Acute induction of seizure may lead to an increased density of A1 receptors (Daval and Werck, 1991) and decreased density of A1 receptor (Ekonomou et al., 2000; Rebola et al., 2003b) or may remain unchanged (Simonato et al., 1994). Furthermore, the changes in the metabolism of adenosine have been observed after repeated seizure induction such as increased release of ATP (Wieraszko and Seyfried, 1989), increased activity of ecto-5′-nucleotidase (Rebola et al., 2003b), or lower density of nucleoside transporters (Pagonopoulou and Angelatou, 1992). These changes may thus result in higher formation and availability of ATP-derived adenosine (Rebola et al., 2003b).

ATP-derived adenosine preferentially activates proconvulsive A2 receptors rather than inhibitory A1 receptors (Cunha et al., 1996). The developmental profile of adenosine A2A receptors reveals low A2A receptor binding at birth and a gradual increase in their density; excess adenosine may enhance this maturation (Doriat et al., 1996). Moreover, the age-related changes in the balance between inhibitory A1- and excitatory A2A-mediated actions have been described. The density of A1 receptor binding sites decreases in the hippocampus, in contrast to the increased number of A2A receptor binding sites in aged rats versus young rats (Cunha et al., 1995). The activation of adenosine A2A receptors decreases presynaptic A1 receptor binding by activation of the protein kinase C—this indicates that the A2A receptors can modulate A1 receptor-mediated signaling. In areas of the brain where A2A receptors are highly expressed, this may be a mechanism by which these two receptors exhibit cross-talk (Lopes et al., 1999). Furthermore, the activation of protein C kinase attenuates A1 inhibition of glutamate release (Barrie and Nicholls, 1993). The activation of A3 receptors by adenosine may also desensitize A1 receptors. A3 receptors are present in rat hippocampal nerve terminal membranes (Lopes et al., 2003).

The changes resulting in an extracellular excess of adenosine may lead to the rapid desensitization of A1 receptors after binding. This, in turn, can lead to A1 receptor downregulation—typically within an hour (Klaasse et al., 2008). This timing of downregulation does not play a role in the early effects of CCPA but might be involved in the late (>1 h) action of the agonist. In contrast, a marked increase of A1 receptor binding sites has been observed within hippocampus only 30 min after the experimental induction of seizure at all developmental stages (Daval and Werck, 1991).

The A2A receptors were also shown to have varying effects ranging from proconvulsant effects to those not detectable or even anticonvulsant effects (Huber et al., 2002). Moreover, in different models of epileptic seizures, activation of A1 receptors also has both proconvulsant and anticonvulsant effects in relation to the mechanism of action of the convulsant used (Klitgaard et al., 1993). Another explanation might be an interaction with other developing neurotransmitter systems. For example, the GABAergic system matures in the third postnatal week in rats when it becomes the main brain inhibitory system (Sanchez and Jensen, 2001).

In the mature brain, GABAA receptors mediate Cl−current flow into the postsynaptic neuron, leading to hyperpolarization and inhibition of neurotransmission. In contrast, repeated seizures result in excessive intracellular Cl−accumulation, and this may cause a transient switch in GABAergic signaling from inhibitory to depolarizing and excitatory (Isomura et al., 2003; Viitanen et al., 2010). A role for the rapid degradation of endogenous adenosine in contrast to the markedly prolonged half-life of A1 receptor agonists should also be considered (Layland et al., 2014). Adenosine is rapidly cleared from plasma with a half-life of 1–2 s (Camm and Garratt, 1991).

Adenosine analogues rapidly increase their half-life due to their resistance to adenosine-metabolizing enzymes (Pavan and Ijzerman, 1998). Unfortunately, there are no data concerning the half-life of CCPA *in vivo*. However, a stability study of the closely related adenosine receptor agonist N^6^-cyclopentyladenosine (CPA) in rat blood was performed (Mathot et al., 1993). The elimination half-life value of CPA in the blood of rats is approximately 25 min (Mathot et al., 1993; Pavan and Ijzerman, 1998).

The literature contains little information about the metabolism of adenosine A1 receptor agonists (Bier et al., 2006), and we are unaware of details on the metabolism of CCPA. Adenosine cannot freely permeate biological membranes, and its transport occurs *via* selected adenosine transporter proteins. Adenosine analogues with a higher half-life may thus be increasingly transported *via* a transport-specific system for adenosine and other purines within the blood–brain barrier (BBB) (Cornford and Oldendorf, 1975). The transport processes are key modulators of extracellular adenosine disposal but have not been characterized extensively. Unfortunately, the transport of CCPA and other A1 receptor agonists within the BBB has not yet been deeply studied.

## Conclusions

The age-dependent anticonvulsant activity of an A1 receptor agonist has been demonstrated in immature brain. The increased expression of A1 receptors at the early stages of development may contribute to the markedly suppressed excitability as demonstrated with the AD thresholds in our experiments. Agonists of A1 receptors might thus be useful as antiepileptic drugs in pediatric neurology.

## Ethics Statement

All procedures involving animals and their care were conducted according to the ARRIVE guidelines https://www.nc3rs.org.uk/arrive-guidelines in compliance with national (Act No 246/1992 Coll.) and international laws and policies (EU Directive 2010/63/EU for animal experiments and the National Institutes of Health guide for the care and use of Laboratory animals (NIH Publications No. 8023, revised 1978). The experimental protocol was approved by the Ethical Committee of the Czech Academy of Sciences (Approval No. 128/2013).

## Author Contributions

All authors listed have made substantial, direct, and intellectual contribution to the work and approved it for publication.

## Funding

This study was supported by grants of the Grant Agency of the Charles University, Second Faculty of Medicine 2120192/2015 and European Regional Development Fund-Projects “PharmaBrain” No. CZ.CZ.02.1.01/0.0/0.0/16_025/0007444, support for long-term conceptual development of research organization RVO: 67985823.

## Conflict of Interest Statement

The authors declare that the research was conducted in the absence of any commercial or financial relationships that could be construed as a potential conflict of interest.
